# Language switches can be monitored but not fully controlled: Eye-tracking evidence for syntax-driven language control

**DOI:** 10.3758/s13423-026-02940-5

**Published:** 2026-07-07

**Authors:** Jessie Quinn, Victor S. Ferreira, Tamar H. Gollan

**Affiliations:** 1 Department of Psychology, University of California, San Diego, 9500 Gilman Dr, La Jolla, CA 92093 USA; 2 Department of Psychiatry, University of California, San Diego, 9500 Gilman Dr, La Jolla, CA 92093 USA

**Keywords:** Language control, Language switching, Eye tracking, Monitoring, Intrusions

## Abstract

When reading aloud mixed-language paragraphs, bilinguals occasionally produce *language intrusions*, that is, they translate written switch words to avoid producing them overtly. Intrusions provide evidence about the nature of *language control*, which refers to how bilinguals maintain control over which language they speak. The present study compared eye movements for successful versus unsuccessful switches during reading aloud of mixed-language paragraphs to reveal the cognitive mechanisms underlying language control failures. Forty-eight English-dominant Spanish-English bilinguals read aloud four stories four times (once each in English-only, Spanish-only, English mixed-language, and Spanish mixed-language conditions). Mixed-language paragraphs included ten language switches (five content words, five function words). Though they elicited the most intrusion errors, dominant-language function (but not content) words elicited more frequent regressive eye movements on successful switches versus intrusions, indicating *heightened* monitoring of the most error-prone switch targets. Additionally, there was no evidence that inattention to dominant-language switches drove their relatively larger elicitation of intrusions – bilinguals skipped and gazed at switch words to similar extents in both languages, and regressed more often to dominant-language than nondominant-language switches regardless of whether they switched successfully or not. These results confirm the vulnerability of dominant-language function words to language control failures, and reveal that these failures occur despite *increased*, but ultimately only partially successful, attempted monitoring. More broadly, these results reinforce proposals that different retrieval mechanisms underlie production of function versus content words, but challenge claims that function words cannot be monitored at the level of planned production prior to production of overt speech.

## Introduction

Bilingual speakers display impressive control over language selection, switching at will but producing few accidental language switches (Gollan et al., 2011; Poulisse & Bongaerts, 1994). However, when they do occur, these errors reflect systematic patterns that provide useful tools for revealing the cognitive mechanisms that allow bilingual speakers to control which language they speak. In spontaneous utterances from 45 native Dutch learners of English, Poulisse and Bongaerts (1994) noted that unintentional language switches most often occurred on function words (i.e., determiners, prepositions, conjunctions, pronouns) compared to content words (i.e., nouns, verbs, adjectives, adverbs; see also Poulisse, 1997). This *part-of-speech effect* is also highly robust in a type of language control failure that occurs occasionally when bilinguals read aloud mixed-language paragraphs. In this task, bilinguals sometimes inadvertently translate a written switch word (Gollan et al., 2014; Kolers, 1966), presumably to avoid switching languages in their speech (e.g., saying “the” instead of “la” when reading “the company was holding auditions in *la* city of London”). Bilinguals produce such intrusion errors even when paragraphs are written mostly in one language (with only a small number of language switches), and the targets of these intrusion errors overwhelmingly involve function rather than content, and dominant rather than nondominant language, switch words (Fadlon et al., 2019; Goldrick & Gollan, 2023; Gollan et al., 2014; Gollan & Goldrick, 2016, 2018, 2019; Quinn et al., 2025; Schotter et al., 2019).

Function words may be more vulnerable to intrusion errors because they are retrieved more automatically, as part of grammatical encoding processes (Garrett, 1990), in contrast to content words which are retrieved at earlier stages during speech production (Bell et al., 2009). However, function and content words vary in multiple ways, many of which could affect how they are processed. In eye-movement studies of silent reading, function words tend to be skipped more than content words due to their higher frequency, predictability and shorter length (Brysbaert et al., 2005; Ehrlich & Rayner, 1981; Just & Carpenter, 1980; Rayner, 1978; Schotter & Dillon, 2025). Even when fixated, function words tend to be processed more efficiently, eliciting shorter gaze durations than content words (O’Regan & Lévy-Schoen, 1987; Rayner & Pollatsek, 1987). There is also evidence that function and content words are processed differently at later processing stages (post-lexically). For example, readers fail to notice when function words are omitted or repeated in a text (e.g., *Amanda jumped off the the swing and landed on her feet*; Healy & Zangara, 2017; Staub et al., 2019, 2024, 2025). In some cases they miss repetitions even when they fixated on *both* instances of the word (Staub et al., 2019).

These robust part-of-speech effects in silent reading raise the possibility that function words might be more prone to intrusion errors in the read-aloud task not because of the difficulty of controlling and monitoring their production, but rather because of reduced attention on those words during reading. A few studies that combined reading aloud of mixed-language paragraphs with eye tracking suggested that intrusions should not be attributed to visual inattention but rather to later processes involving speech planning and monitoring (Gollan et al., 2014; Ratiu & Azuma, 2017; Schotter et al., 2019). Gollan et al. (2014) found that skipping switch words increased intrusion rates, but bilinguals produced the most intrusion errors even when they fixated on the switch word. Ratiu and Azuma (2017) found no difference in gaze durations when bilinguals intruded compared to when they switched successfully, while Schotter et al. (2019) found shorter gaze durations on words produced as intrusions, but this was equally true for content and function switches. Additionally, bilinguals regressed back to content switches that elicited intrusion errors significantly more often when they self-corrected the error versus when they did not. However, regressing back to switches did not facilitate self-repairs of errors on function words, possibly implying that function words elicit more intrusion errors because it is difficult, perhaps even impossible, to monitor and stop their production once it has been planned.

Another robust driver of intrusion errors during reading aloud is language dominance. Most intrusions occur on dominant-language switch words, often leading to an apparent *reversal* of language dominance during the task (e.g., Fadlon et al., 2019; Goldrick & Gollan, 2023; Gollan et al., 2014; Gollan & Goldrick, 2016, 2018; Li & Gollan, 2018; Quinn et al., 2025; Schotter et al., 2019; Stasenko et al., 2021). Dominance reversal is thought to be a product of inhibition of the dominant language during production of the nondominant language, which in turn makes it more difficult to switch into the dominant language (for recent discussion, see Goldrick & Gollan, 2023). Alternatively, a bilingual’s dominant language might be more susceptible to intrusion errors while reading not because of inhibition of dominant-language representations during nondominant-language reading, but rather due to increased efficiency when processing words in the dominant compared to the nondominant language (i.e., more skipping, shorter gaze durations, and fewer regressions; e.g., Cop et al., 2015; Dirix et al., 2020). No studies have investigated language dominance in eye-tracking measures during reading aloud even though dominance greatly affects the production of intrusion errors.

### Current study

The present study investigated the role of attention and monitoring for maintaining production of the intended language by examining eye movements to switch words, contrasting successful versus unsuccessful switches to function versus content words and dominant versus nondominant language switches. Schotter et al. (2019) did not include correctly produced switches in their analysis of regressions, and only analyzed intrusions in the dominant language (Chinese-English bilinguals in that study rarely produced intrusions on nondominant language targets, insufficient for analysis). Thus, it remains unclear if regressions to switch words might facilitate the production of a successful switch and if monitoring efforts might reduce errors for both function and content switch words. Even less is known about language-dominance effects, which have not been fully considered in conjunction with eye-tracking evidence.

Forty-eight Spanish-English bilinguals read aloud four paragraphs four times, once in each of four different conditions (English-only, English-mixed, Spanish-only, Spanish-mixed) while their eyes were tracked. We hypothesized that if monitoring facilitates language switching, bilinguals should show more regressions to switch words when switching successfully compared to when they produce an intrusion error. Of particular interest is whether bilinguals would show any evidence of monitoring of the most intrusion-prone switch targets, that is, dominant-language function words. If so, regression rates to these words should be significantly greater when switching successfully versus intruding. In line with previous studies (e.g., Gollan et al., 2014; Schotter et al., 2019), we expected that inattention to switch words – operationalized as more skips and shorter gaze durations – would increase the production of errors versus successful switches. Importantly, if dominant-language function words are especially vulnerable to intrusions because of inattention, we expected more skips and shorter gazes for intrusions versus successful switches, especially for dominant-language function words.

## Method

### Participants

Forty-eight English-dominant Spanish-English bilinguals were recruited through undergraduate classes at the Department of Psychology, University of California, San Diego, and either received course credit or $15 compensation for their participation. Language dominance was classified based on the Multilingual Naming Test (MINT) Sprint 2.0 (Gollan et al., 2024; Neveu et al., 2025; see also Garcia & Gollan, 2022). Participants who did not name at least five more pictures in English than in Spanish were replaced (to ensure a robust manipulation of language dominance). Participant characteristics are shown in Table 1. The majority of participants learned Spanish as their first language and acquired English later, eventually all becoming dominant in English. All participants gave their informed consent prior to the study.

**Table 1:** Participant characteristics

Characteristic	*M*	*SD*
Age	20.6	2.3
Years of college education	2.5	2.0
Age of English acquisition	3.7	2.6
Age of Spanish acquisition	0.6	1.0
English total MINT Sprint 2.0 score^a^	67.6	5.3
Spanish total MINT Sprint 2.0 score^a^	46.5	8.3
Percent English use in childhood	28.2	24.6
Percent English use currently	64.5	35.2

MINT = Multilingual Naming Test

^a^ Number of pictures named correctly out of 80 total

### Materials and procedure

Four paragraphs that elicited higher rates of intrusion errors in Quinn et al. (2025) were edited for the present study. Paragraphs appeared in four conditions: single-language English only, single-language Spanish only, mixed-language English default, mixed-language Spanish default – here *default language* refers to the majority language of the paragraph. Paragraphs averaged 197 (*SD = *6.11) words in length. Each paragraph contained ten target words that were chosen based on higher intrusion rates in previous studies (Fadlon et al., 2019; Gollan & Goldrick, 2018; Quinn et al., 2025): five function words and five content words. Target words were noncognates and all content targets were nouns. Target words were dispersed throughout the paragraph and never appeared at the beginning or end of a sentence or next to punctuation. Additionally, targets never appeared as the first or last word of a line on the computer screen. In mixed-language paragraphs, target words switched from the default language to the other language while in single-language paragraphs target words were in the same language as the rest of the paragraph. The same five function word targets (*but/pero, and/y, the/la, because/porque, that/que*) appeared across all four paragraphs. Two sets of five content words were also repeated (and appeared as switch words in two different paragraphs/stories). One set of content words (*life/vida, woman/mujer, water/agua, step/paso, months/meses*) repeated across two paragraphs and another set (*father/padre, beach/playa, friends/amigos, fish/peces, sun/sol*) repeated across the other two paragraphs.

Bilinguals were randomly assigned to one of 16 lists. List number determined the presentation order of each paragraph. Bilinguals always read all four versions of a paragraph in a row (e.g., English-only, Spanish-only, English-mixed, Spanish-mixed). Half read both single-language versions of the paragraph before reading both mixed-language versions, and half read both mixed-language versions before the single-language versions. The language of the first presentation of a paragraph was counterbalanced within and between participants and paragraph default language changed with each repetition (i.e., participants never read the same paragraph in the same default language in a row). Before reading the experimental paragraphs, participants read two practice mixed-language paragraphs. None of the switch words in the practice paragraphs were the same as the experimental paragraphs.

After completing the read-aloud task, participants completed a Language History Questionnaire before completing the MINT Sprint 2.0. The entire procedure took about 60 minutes to complete.

### Apparatus

Participants’ right eye movements were recorded with an Eyelink 1000 Plus (SR Research Ltd., 2013) at a sampling rate of 1,000 Hz. Paragraphs were displayed in 30-point Calibri font with 1.5 line spacing on a 1,920 x 1,080 Asus VG248 monitor. Participants were situated approximately 115 cm away from the display monitor and rested their forehead on the headrest to minimize head movement while still allowing for language production (the chin-rest was removed). Before beginning the experiment, calibration and validation were performed with a 9-point grid, and the tracker was recalibrated any time error exceeded 0.5 degrees of visual angle. On average, recalibration was performed twice throughout the task.

### Data analyses and models

For all analyses below we included only switch words in mixed-language paragraphs (single-language paragraphs do not elicit intrusion errors). Data were analyzed using R Statistical Software (Version 4.5.3; R Core Team, 2026) and the packages *lme4* (Version 1.1.38; Bates et al., 2015) and *emmeans* (Version 2.0.1; Lenth, 2025). Models included contrast-coded fixed effects and their interactions. This included language of the switch word (.5 English, -.5 Spanish), part of speech (.5 content, -.5 function), and following Schotter et al. (2019) for analyses of eye movements, we contrast coded production type (.5 successful switch, -.5 intrusion).^1^ To account for word-length differences across part of speech, all models included word length centered as a non-interacting covariate. Lastly, all models included random intercepts for subject and word. Models with more complex random effects structures failed to converge or showed a singular fit. Likelihood ratio tests were performed to obtain *p-*values for all fixed effects. 

We first analyzed intrusion rates by language and part of speech. Analyses of eye movements included trials in which participants switched successfully or produced an intrusion, but excluded partial intrusions (which can be more subjective and difficult to code; Gollan & Goldrick, 2016) and late corrections (which occurred less than uncorrected intrusions, see Table 2, and arguably represent a middle-of-the-road level of attention and monitoring, which would blur the distinctions of primary interest). Our eye-tracking measures of interest were *skip rate* – the proportion of target words not fixated during the first-pass, an index of overt attention; *gaze duration* – the total time spent fixating on the target word during the first-pass, an index of attention and lexical accessibility; and *regression rate* – the proportion of targets that were fixated following a fixation of a word further in the paragraph, to index monitoring.

**Table 2:** Counts of production type by language and part of speech of switch word

Production type	Language	Function	Content
Successful switch	English	613	847
	Spanish	858	936
All intrusions	English	310	98
	Spanish	74	11
Full intrusion	English	223	45
	Spanish	44	3
Late correction	English	66	16
	Spanish	28	2
Partial intrusion	English	21	37
	Spanish	2	6

Full intrusions were those which were never corrected. Late corrections occurred when participants corrected their intrusion after production. Partial intrusions were those corrected mid-production and are included here for reference but were not analyzed. Participants produced other error types (e.g., within-language errors), but these are not shown here

Prior to eye-movement analyses, gaze durations less than 50 ms were removed and not analyzed (following Schotter et al., 2019). This excluded less than 1% of the data. For gaze-duration analyses, we removed all words skipped during the first pass. Regressions back to target words included both initially fixated and skipped words, and the baseline for regression rate included all words that were and were not fixated (i.e., we changed the default Eyelink Data Viewer values for words never fixated from NAs to 0 s so they would be included in the denominator as words not regressed to). Logistic mixed-effects models analyzed regression and skip rate and linear mixed-effects models analyzed gaze duration.

## Results

### Intrusion errors

Table 2 shows counts of successful switches and intrusions produced on switch words and Table 3 shows intrusion rates by part of speech and language. Table 4 shows the model outputs. Replicating previous studies, bilinguals produced more intrusion errors on dominant-language than nondominant-language words (χ^2^(1) = 14.66, *p* <.001) and function than content (χ^2^(1) = 6.97, *p* =.008) words. The interaction was not significant.

**Table 3:** Intrusion rates by language and part of speech of switch word. Standard deviations are given in parentheses

Language	Part of speech	Intrusion rate
English	Content	.06 (.24)
	Function	.30 (.46)
Spanish	Content	.01 (.07)
	Function	.07 (.26)

**Table 4:** Intrusion error model outputs

Fixed effect	β	s.e. β	χ^2^	*p*
**Part of speech**	**−2.06**	**0.78**	**6.97**	**.008**
**Language**	**3.07**	**0.73**	**14.66**	**<.001**
Word length	−1.13	0.60	3.27	.07
Part of speech x Language	−0.26	1.40	0.03	.85

### Eye-movement measures

Following Schotter et al. (2019), we focused our analyses on those conditions where the number of observations exceeded 28 (see Table 2); specifically, we did not analyze Spanish content words given that bilinguals produced few intrusion errors in this condition. Because there were not enough data points for a single analysis crossing all factors, we instead completed two analyses to address the vulnerability of English function words to intrusions – first we compared them to English content words, then we compared them to Spanish function words, analyzing part-of-speech and language effects separately.

**Fig. 1:**
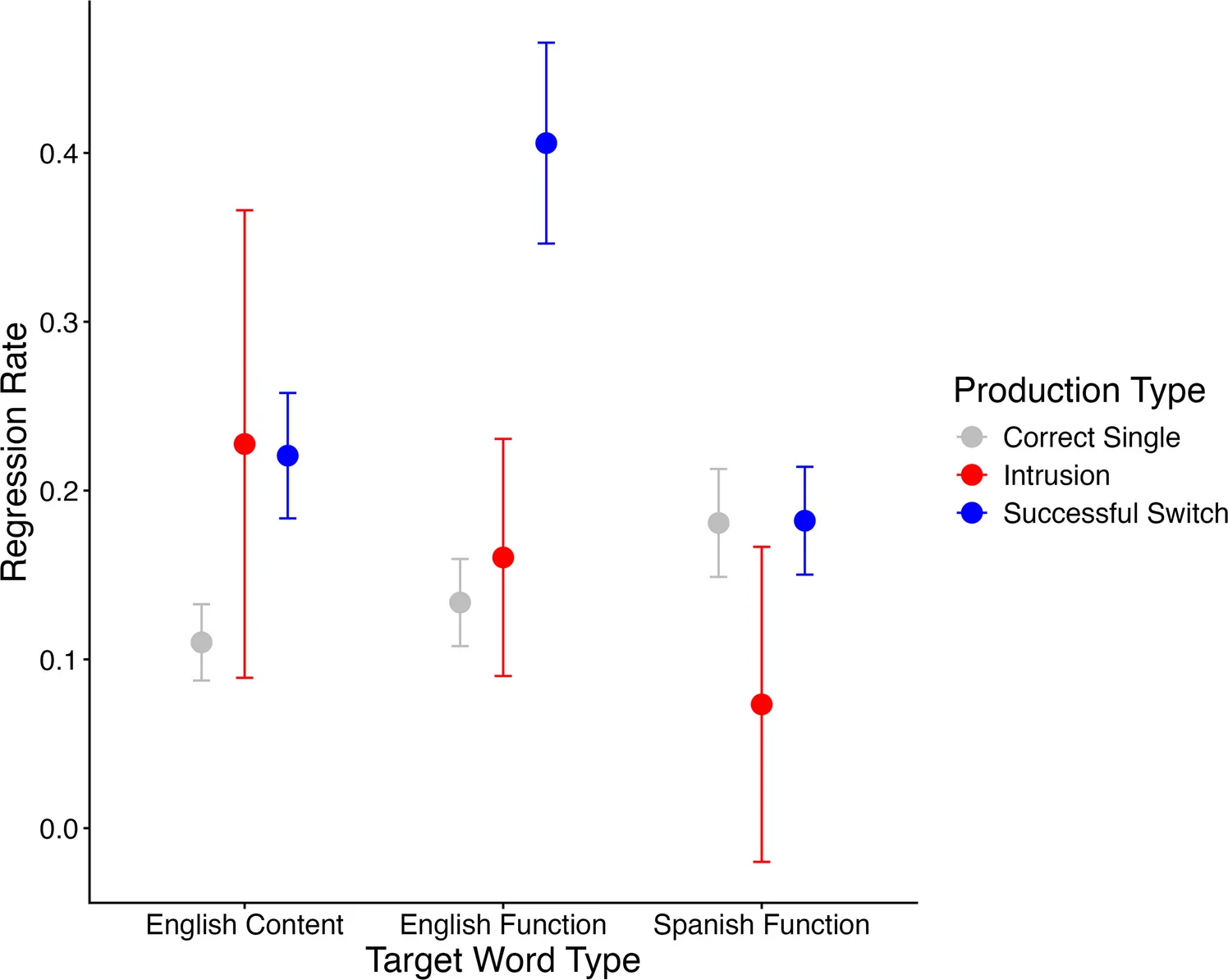
By-subject mean regression rates to target words by production type and target word type. Error bars reflect 95% confidence intervals. Bilinguals regressed to English function words significantly more when switching successfully (in blue) compared to when intruding (in red). Regression rates on target words without a language switch (in single-language paragraphs) are shown in gray for comparison (these targets did not elicit intrusion errors)

Figure 1 shows average regression rates and Table 5 shows average skip rates and gaze durations. Both include target words correctly produced in single-language paragraphs as a reference point, even though these were not included in the analyses. Table 6 shows model outputs for the following analyses.

**Table 5:** By-subject mean gaze durations and skip rates by production type, language, and part of speech. Standard deviations are given in parentheses

Measure	Production type	Language	Function	Content
Gaze duration	Successful switch	English	332 (88)	349 (71)
		Spanish	325 (57)	322 (67)
	Intrusion	English	293 (95)	283 (176)
		Spanish	230 (45)	288 (244)
	Correct single	English	298 (45)	299 (52)
		Spanish	326 (60)	300 (54)
Skip rate	Successful switch	English	.32 (.42)	.19 (.11)
		Spanish	.35 (.13)	.17 (.12)
	Intrusion	English	.48 (.33)	.22 (.37)
		Spanish	.63 (.45)	.33 (.58)
	Correct single	English	.42 (.14)	.25 (.13)
		Spanish	.44 (.12)	.18 (.09)

**Table 6:** Model outputs for eye measures

Analysis	Measure	Fixed effect	β	s.e. β	χ^2^	*p*
Part of speech	Skip rate	Part of speech	−0.62	0.37	2.63	.11
		Production type	−0.22	0.23	0.90	.34
		**Word length**	**−0.82**	**0.26**	**7.35**	**.007**
		Part-of-speech x Production type	0.26	0.46	0.33	.57
	Gaze duration	Part of speech	−2.21	30.61	0.01	.94
		**Production type**	**61.86**	**21.39**	**8.32**	**.004**
		Word length	−8.69	20.57	0.18	.67
		Part of speech x Production type	41.74	42.79	0.95	.33
	Regression rate	Part of speech	−0.21	0.20	0.30	.58
		**Production type**	**0.80**	**0.23**	**13.26**	**<.001**
		Word length	−0.36	0.26	1.75	.19
		**Part of speech x Production type**	**−1.34**	**0.45**	**8.01**	**.005**
Language	Skip rate	Language	0.42	0.24	3.16	.08
		Production type	−0.15	0.20	0.60	.44
		**Word length**	**−1.31**	**0.14**	**25.42**	**<.001**
		Language x Production type	−0.18	0.39	0.22	.64
	Gaze duration	Language	41.38	25.45	2.60	.11
		**Production type**	**82.02**	**24.45**	**11.11**	**<.001**
		Word length	−0.56	10.04	0.003	.96
		Language x Production type	−69.02	47.58	2.08	.15
	Regression rate	**Language**	**1.12**	**0.33**	**11.25**	**<.001**
		**Production type**	**1.30**	**0.30**	**25.87**	**<.001**
		**Word length**	**−0.33**	**0.14**	**4.64**	**.03**
		Language x Production type	0.39	0.59	0.41	.53

#### Part of speech

Bilinguals skipped shorter more than longer words (λ^2^(1) = 7.35, *p* =.007), gazed at switch words longer (λ^2^(1) = 8.32, *p* =.004) and regressed to switches more (λ^2^(1) = 13.26, *p* <.001) when they switched successfully than when they produced intrusions. Of great interest, bilinguals regressed back to function (*β* = −1.46, *p* <.0001) but not content switches (*β* = −0.13, *p* =.74) more often when they switched successfully versus when they produced intrusions, an interaction between production type and part of speech (λ^2^(1) = 8.01, *p* =.005).

#### Language

Bilinguals skipped (λ^2^(1) = 25.42, *p* <.001) and regressed to (λ^2^(1) = 4.64, *p* =.03) shorter more than longer words. Bilinguals gazed at switches longer (λ^2^(1) = 11.11, *p* <.001) and regressed to them more often (λ^2^(1) = 25.87, *p* <.001) when they switched successfully than when they produced intrusions. Bilinguals also regressed to dominant-English switches more often than nondominant-Spanish switches (λ^2^(1) = 11.25, *p* <.001), but equally often when producing successful switches versus intrusion errors (no interaction between language and production type).

### Regression and production-onset interval

To aid interpretation of regressive fixations to language switches on English function words we asked whether these occurred prior to or after the onset of their production. We assumed that regressions that occurred prior to speech onset reflect monitoring of planned speech and facilitated successful switches. By contrast, regressions that occurred after speech onset might reflect a different form of monitoring (i.e., checking if an error was produced). To this end, we subtracted production-onset times of correct switches from the start time of the regressive fixation back to the switch word (negative values indicate that the regressive fixation happened prior to, and positive values after, production onset).^2^

**Fig. 2:**
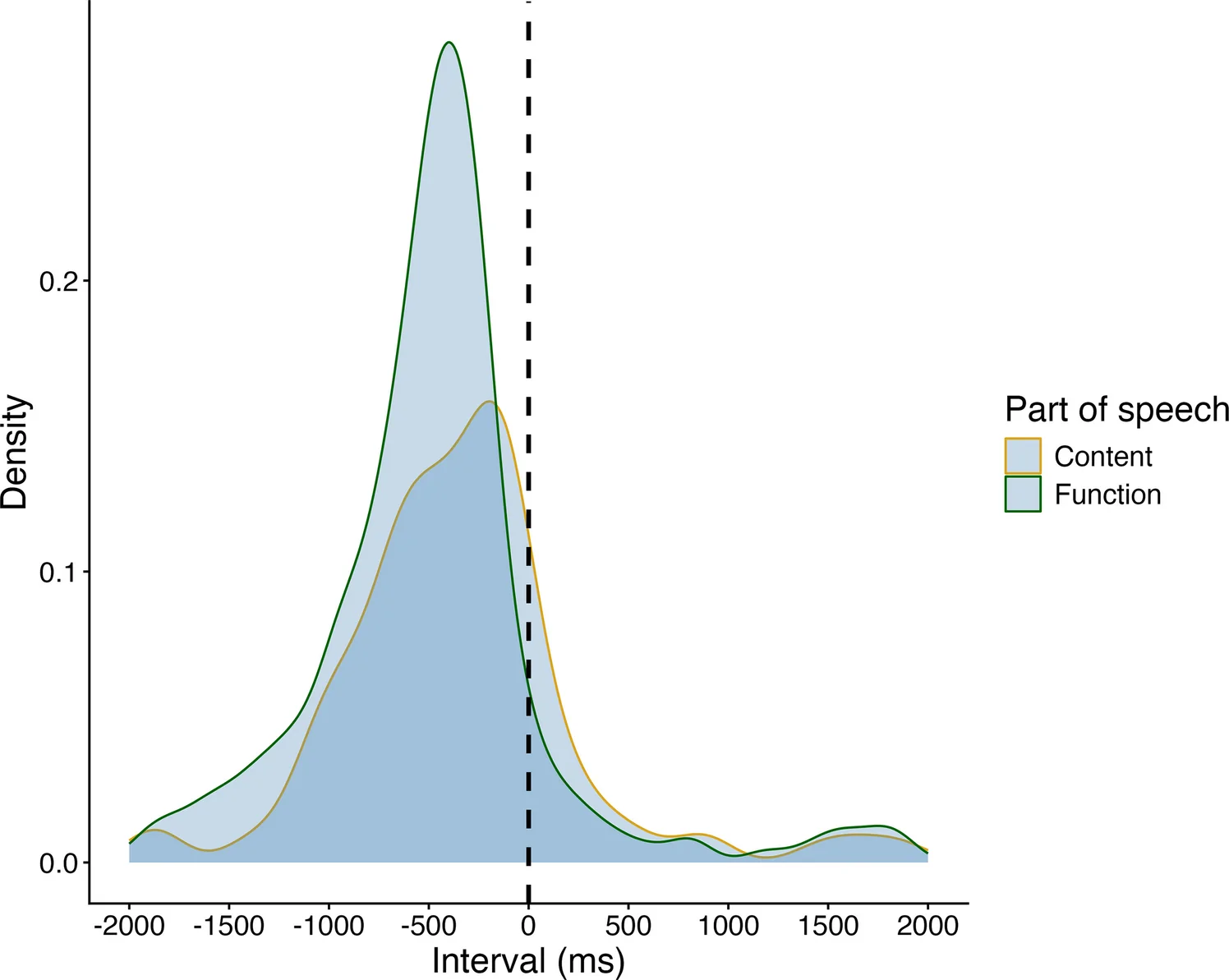
Distribution of the production onset and regressive fixation interval for correctly produced English switch words. They-axis reflects probability densities such that the probability of a value falling between a given interval is equal to the area under the curve for that interval. Negative values on the x-axis indicate that the regressive fixation to the target switch word occurred prior to the production onset of the switch word

Shown in Figure 2, in the overwhelming majority of cases, regressions to correctly produced English switch words occurred prior to their production; 88% of function (211/239; *M* = −430 ms, *SD* = 402) and 82% of content switches (148/180; *M* = −235 ms, *SD* = 592). Critically, when removing regressions that occurred after the switch onset, the interaction between production type and part of speech in the analysis of regressions to English switch words remained significant (β = −1.17, *SE* β = 0.50, χ^2^ = 4.88, *p* =.03).

## Discussion

The results of the present study confirmed that dominant-language function words elicit more intrusion errors in the read-aloud task and provide evidence that inattention and monitoring failures *during reading* do not underlie this vulnerability. Though dominant-language function words were most intrusion prone, this occurred *despite significantly increased* regressions to these target words before their successful production. Additionally, bilinguals skipped function word switches in both languages equally often, gazed at function word switches in both languages equally long, and regressed to dominant-English more than nondominant-Spanish function switch words regardless of production type. Finally, bilinguals gazed at switch words longer when they switched successfully versus when they produced intrusions, but this did not interact with part of speech (replicating Schotter et al., 2019), and bilinguals skipped short words more often than long words, but this did not vary by production type, part of speech or language.

### Dominant-language function words are intrusion-prone despite increased monitoring

English function words elicited between four to 30 times as many intrusions as other targets (see Table 3) but this was clearly not because of an inability, or because of reduced attempts, to monitor these switches. Figure 1 shows bilinguals regressed to about 40% of English function switches when they produced them correctly, about twice the regression rate to successful switches for English content and Spanish function words. Moreover, Figure 2 shows that the majority of these regressions occurred prior to the onset of production of the switch word, which suggests that regressions facilitated correct production of the most difficult to produce language switches. This seems inconsistent with proposals that errors on function words are effectively “never seen” (Staub et al., 2019, 2024), and with our prior conclusion that intrusions on function words cannot be stopped once they have been planned (Schotter et al., 2019). Monitoring might have been too cognitively demanding in Schotter et al. (2019) given that nearly half of the words in the paragraphs were language switches (note that conditions with greater interference should lead to higher error detection rates according to Nozari et al., 2019, but perhaps this collapses when the system is overloaded). Similarly, longer gaze durations on words produced as successful switches might also reflect heightened control and monitoring; however, it seems that only function switches required, or benefitted from, additional processing (via regressions) to switch successfully.

Independent evidence links regressions to monitoring or similar processes (e.g., Bicknell & Levy, 2011; Booth & Weger, 2013; Inhoff et al., 2019; Schotter et al., 2014, 2019). After initially processing the target switch during the first pass (either in the periphery for skipped words or after fixating and leaving the target), bilinguals executed fast regressive saccades to re-examine function switches on average 430 ms prior to the onset of their successful production. These regressions may reflect internal monitoring within the production system which is triggered by response conflict (e.g., Nozari et al., 2011) and allows for rapid repairs of planned errors prior to their articulation. Consistent with this view, bilinguals detected and repaired more of their errors on language switch trials, where conflict is high, compared to nonswitch trials (Declerck et al., 2017).

Conflict could arise when readers generate predictions about upcoming text (and execute the speech plan for these predictions) which then fail to match the incoming stimulus, triggering both increased errors and initiating the processes needed to repair them. Function word switches might create more conflict than content words because they are more predictable (Quinn et al., 2025; but see Gollan et al., 2024). On this view, more control resources are needed to enhance recognition and/or planned production of function word switches via regressive saccades as a way of “checking” that planned speech matches the written words. Interestingly, partial intrusions more often involved content than function words (see Table 2; also Gollan et al., 2014; Gollan & Goldrick, 2016; Schotter et al., 2019). Speech errors that are corrected mid-production (Levelt, 1983, 1989, Levelt et al., 1999) are corrected too quickly to have been caught by an external monitor and, thus, must have been detected internally but repaired slightly after articulation onset (Nooteboom & Quené, 2017, 2020). Curiously, function words may rarely lead to partial intrusions not because they are rarely caught early, but because the production of high-frequency and often short function words is more ballistic – and so if an impending function word error is not caught before the initiation of articulation (here, often after a regressive eye movement), it will not get caught at all. Content words, in contrast, are lower frequency and longer and so more able to be halted mid-word. Further insight as to how control mechanisms differ by part of speech might be gained by contrasting reading aloud with purer production tasks where conflict arises internally rather than from a mismatch between an internal speech plan and an external stimulus.

### Intrusion errors in the read-aloud task arise during language production not visual word recognition

Bilinguals gazed at switch words less when producing intrusion errors versus successfully switching. However, we found no evidence that dominant-language switches elicited more intrusions because they were less attended than nondominant-language switches (see also Gollan et al., 2014; Schotter et al., 2019). Instead, switching on dominant-language words may reflect control mechanisms engaged during language production. Previously we suggested that speaking in the nondominant language may elicit proactive inhibition over the dominant language to reduce potential interference. Inhibition might occur at the whole-language level especially to suppress automatic retrieval processes, including the retrieval of function words (Gollan & Goldrick, 2018; Quinn et al., 2025). Such inhibition may help bilinguals avoid interference from very frequent dominant-language function words, but is clearly quite difficult to overcome when the task demands inhibition be released. This might explain why high frequency, dominant-language function words are paradoxically less accessible as switch targets when the nondominant language is selected for production. In a pure production task, language dominance effects, and the inhibition needed to control them, might be even stronger since activation of the nondominant language would not be boosted by written stimuli (Gollan et al., 2011; Gavino et al., 2025).

## Conclusion

Our results provide new insights which suggest that the most intrusion-prone targets in the read-aloud task are vulnerable despite increased attempts to monitor them. This confirms previous conclusions that robust part-of-speech effects on bilingual language control do not reflect reduced attention, extends them to include language-dominance effects, and provides evidence that function words can be internally monitored and corrected prior to the production of errors. More broadly, these results support models of language production that assume function words are retrieved via different underlying cognitive mechanisms than content words, both highlighting their relative automaticity and also revealing some conditions under which their production *can* be monitored and better controlled.

## Data Availability

All data and materials are publicly available on the Open Science Framework at: https://osf.io/zpsg8

## Footnotes

1 Models including repetition as a fixed and interacting factor showed bilinguals produced more intrusions for content and function words with repetition. Critically, however, including repetition did not improve model fit in any analysis of eye-movement data (AIC values were always lower for the simpler models). Paragraph repetition was therefore excluded from all final models.

2 For trials where participants regressed to a correctly produced English switch word, we extracted production-onset times by first aligning the experimental audio files with the participants’ transcribed production data using the Montreal Forced Aligner (MFA; McAuliffe et al., 2017), which can handle English and Spanish data. The MFA outputs Praat (Boersma & Weenink, 2026) TextGrid files for each paragraph which we used to extract onset times for each target switch using the rPraat package (Bořil & Skarnitzl, 2016) in R. In the eye-tracking fixation reports, we identified whether a fixation was regressive by comparing its interest area ID to that of the fixation that occurred before it. For fixations where the previous fixation ID was greater than the current (i.e., the previous fixation was on a word further along in the paragraph), we extracted the fixation start time as the start of the regression. We subtracted the production-onset time from the start of the regression to get the interval between the regressive fixation and the production onset.

## Code availability

The analysis code is publicly available on the Open Science Framework at: https://osf.io/zpsg8
